# Appraisal of phytochemical and in vitro biological attributes of an unexplored folklore: *Rhus Punjabensis* Stewart

**DOI:** 10.1186/s12906-017-1659-6

**Published:** 2017-03-09

**Authors:** Saira Tabassum, Madiha Ahmed, Bushra Mirza, Muhammad Naeem, Muhammad Zia, Zabta Khan Shanwari, Gul Majid Khan

**Affiliations:** 1 Department of Biotechnology, Faculty of Biological Sciences, Quaid-i-Azam University Islamabad, Islamabad, 45320 Pakistan; 2 Department of Pharmacy, Faculty of Biological Sciences, Quaid-i-Azam University Islamabad, Islamabad, 45320 Pakistan; 3 Department of Biochemistry, Faculty of Biological Sciences, Quaid-i-Azam University Islamabad, Islamabad, 45320 Pakistan

**Keywords:** HPLC-DAD, Antioxidant, Brine shrimps cytotoxicity, Anticancer, Antileishmanial

## Abstract

**Background:**

The role of plants for discovery of therapeutic potential accentuates the need to know their biological attributes. The present study aims to comprehend the biological attributes of *Rhus punjabensis,* an unexplored traditional medicinal plant.

**Methods:**

Leaf and stem extracts of *R. punjabensis* prepared in 11 different organic solvents are evaluated for multimode antioxidant potential, total phenolic and flavonoid contents were determined through colorimetric assays, HPLC-DAD analysis was carried out for quantification of various polyphenols in extracts. Brine shrimp lethality, SRB and MTT assays were used to elucidate plant’s cytotoxic and antileishmanial potentials. Disc diffusion assay was used to elucidate the protein kinase inhibitory, antibacterial and antifungal spectrum.

**Results:**

Ethanol + ethyl acetate yielded maximum extract recovery from leaf (6.11 ± 1.09% w/w), total phenolic content (80.5 ± 2.18 μg GAE/mg extract) and reducing power potential (165.4 ± 2.29 μg AAE/mg extract). Maximum flavonoid content (30.50 ± 1.11 μg QE/mg extract) and highest DPPH based free radical scavenging activity (IC_50_ 11.4 ± 2.07) was exhibited by the methanol + chloroform leaf extract. The methanol extract showed maximum total antioxidant capacity (74.5 ± 2.25 μg AAE/mg DW), protein kinase inhibitory (12.5 ± 1.10 bald phenotype at 100 μg/disc) and antifungal (MIC = 25 μg/disc against *Aspergillus flavus*) potential. Reverse phase HPLC-DAD based quantification reveals presence of gallic acid, apigenin, rutin and catechin in various extracts. Brine shrimp lethality assay demonstrated most extracts as highly cytotoxic (LC_50_ < 50 μg/mL) whereas chloroform extract of leaf demonstrated maximuminhibition against human leukemia cell line (IC_50_ 7.80 ± 0.01 μg/mL). A significant activity against leishmanial promastigotes was demonstrated by n-hexane leaf extract (IC_50_ = 15.78 ± 0.15 μg/mL). A better antibacterial activity,by the extracts, against Gram positive strains as compared to Gram negative was observed.

**Conclusions:**

Results recommend multiple-solvent system as a critical factor to sumptuous the biological prospective of *R. punjabensis* and propose it to be a useful natural hub for the discovery of novel antioxidant, anticancer, antileishmanial and antimicrobial agents.

## Background

The exceptionality in structural diversity of Mother Nature has been the most imperative source of novel biomarkers against a number of unremitting and ever more challenging health anomalies since antiquity. As the plant inhabitant secondary metabolites are evolved within living systems, they are often alleged to possess greater drug likeness and biological friendliness. Therefore, there has been a tremendous surge in the phytochemical and biological prospecting of ethno-medicinal herbal remedies nowadays.

The genus *Rhus* encompassing around 250 species of flowering plants is a well acknowledged ethno-medicinal genus which is extensively used by the local people of Pakistan especially in the Khyber PakhtoonKhwa (KPK) province. It belongs to family Anacardiacaea; comprising of mostly small trees and shrubs, often with a resinous bark and a milky sap, leaves are alternate, simple or compound, flowers are bisexual or unisexual [1]. Sumac is the common name for *Rhus*, the name is originated from “sumaga” meaning red in Syriac and is recognized to possess both nutritional as well as medicinal importance [2]. *Rhus* normally grows in non-agricultural regions and is used by indigenous people as food as well as medicinal and other purposes [3]. *R. punjabensis* Stewart and the allied species of *Rhus* are widely used in the folk medicine to treat hepatitis and other ailments in the local vicinity of Karak, KPK Pakistan, however scientific literature about this specie is dearth. In traditional system of medicines the *Rhus spp*. has been used in the treatment of diarrhea, dysentery, ulcer wound healing, leucorrhea, sore of throat, conjunctivitis, ophthalmia, diuresis, animal bites, pain, poison, and liver disease [4]. Bark cocktail is used in treatment of viral eye infections and also applied on forehead as first-aid treatment of epitasis [5]. Powdered fruits are sprinkled on boiled egg and eaten for the treatment of diarrhea [6]. The decoction of fruits is administered orally for the treatment of diarrhea and urinary system disorders as well as liver diseases [4]. Despite of the several claims for other species, scientific data about the biological profile of *R. punjabensis*is still in paucity*.* To the best of our knowledge this is the first comprehensive report that has unveiled the phytochemical, antioxidant, protein kinase inhibitory, anticancer, antileishmanial and antimicrobial prospective of the subject plant*.* A range of solvent systems of escalating polarity has been employed to determine the best solvent system for each type of bioactivity. In contrast to the previous studies employing one or two solvent extracts which do not possibly dissolve all phytochemicals, the current study is designed to evaluate the hidden potential of different solvent soluble extracts with reference to the polarity of active compounds present in the herb of *R. punjabensis.*

## Methods

### Collection and authentication

*R. punjabensis* Stewart was collected in July 2013 from Lakary mountain, Shamshaki, District Karak, Khyber Pakhtunkhwa, Pakistan. The collection site was wild and did not require authorization from any regulatory agency. The plant material was authenticated by Prof. Dr. Mir Ajab Khan, Department of Plant Sciences, Quaid-i-Azam University Islamabad, Pakistan. A voucher specimen HMP 491 was deposited in the Herbarium of Medicinal Plants, Quaid-i-Azam University Islamabad, Pakistan.

### Chemicals and reagents

The study involves use of analytical grade solvents i.e. n-hexane; chloroform; acetone; ethyl acetate; ethanol; methanol and dimethyl sulfoxide (DMSO) purchased from Sigma (Sigma-Aldrich, USA). The reagents include gallic acid; quercetin; 2,2-diphenyl-1-picryl-hydrazyl (DPPH); potassium acetate; aluminum chloride; Folin-Ciocalteu (F-C) reagent; sodium carbonate; ascorbic acid; ammonium molybdate; sodium phosphate; sulfuric acid; ferric cyanide; trichloroacetic acid and potassium ferricyanide procured from Merck (Merck KGaA, Germany), rutin; myricetin; caffiec acid;apigenin;kaempferol; catechin; and caffeic acid were purchased from Sigma (Sigma-Aldrich, Germany). Sabouraud dextrose agar (SDA), nutrient broth and nutrient agar media were purchased from Merck (Merck KGaA, Germany). RPMI-1640, fetal bovine serum (FBS) and medium 199 were purchased from Sigma (Sigma Chemical Co., St. Louis, MO). Equipment included Agilent 1200 series binary gradient HPLC coupled with diode array detector (Agilent Technologies, Germany); CO_2_ incubator (mco-17AIC,Sanyo-Japan); improved neubauer chamber (Marien, Germany). Reference standards employed in the current study, werepurchased from Sigma-Aldrich USA, include droxithromycin; cefixime; terbinafine; doxorubicin; surfactin; amphotericin-B; 5-Florouracil and vincristine.

### Preparation of extracts

*R. punjabensis* leaves and stem were rinsed with water and dried under shade at room temperature. After comminuting to coarse powder by commercial grinder, 15 g powder was soaked separately in beakers, each containing 45 mL of extraction solvents. A variety of solvents were applied ranging from non-polar to highly polar including *n*-hexane (*n*-hex), chloroform (CHCl_3_), acetone (Acet), ethyl acetate (EtOAc), ethanol (EtOH), methanol (MeOH), ethanol + chloroform (EtOH + CHCl_3_; 1:1), methanol + chloroform (MeOH + CHCl_3_; 1:1), acetone + ethyl acetate (Acet + EtOAc; 1:1), ethanol + ethyl acetate (EtOH + EtOAc; 1:1); and methanol + ethyl acetate (MeOH + EtOAc; 1:1). After 3 days of soaking, solvent was filtered through Whatman no.1 filter paper and the residue was dipped again in respective solvent and filtered again thereafter. The solvents were combined and concentrated under reduced pressure in a rotary evaporator (Buchi, Switzerland) at 45 °C. Percentage yield of extracts was calculated gravimetrically.

### Phytochemical screening

#### Determination of total phenolic contents (TPC)

Total phenolic contents were determined using standard protocol [7]. In brief, stock solutions (4 mg/mL) of the extracts were prepared in DMSO and 20 μL of each was transferred to 96 well plate. Folin-Ciocalteu reagent (90 μL) was added to each well and after 5 min 90 μL Na_2_CO_3_ (7.5% w/v in H_2_O) was mixed in each well. The reaction mixtures were incubated for 1 h and absorbance was measured at 630 nm using microplate reader (Bioteck, USA). Blank (DMSO) and standard (gallic acid in DMSO) were run simultaneously as control. A calibration curve (y = 0.0135x + 0.0846, R^2^ = 0.986) was obtained in parallel under the same experimental conditions using gallic acid (6.25–50 μg/mL). The resultant TPC is determined as μg gallic acid equivalent per mg dry weight (μg GAE/mg extract).

#### Determination of total flavonoid content (TFC)

Total flavonoid contents were determined according to the method previously described [8]. Briefly 20 μLof sample (4 mg/mL), 10 μL of aluminium chloride (10% w/v in H_2_O), 10 μL of 1.0 M potassium acetate and 160 μLof distilled water were added in 96 well plate which was incubated at room temperature for 30 min. The absorbance of the plate was measured at 415 nm using microplate reader (Bioteck, USA). The calibration curve (y = 0.0269x + 0.00765, R^2^ = 0.998) was drawn by using quercetin as standard at 0 to 40 μg/mL and the flavonoid content were established as μg quercetin equivalent per mg plant extract (μg QE/mg extract).

### Quantitative evaluation by HPLC-DAD

HPLC-DAD analysis of the extracts for polyphenols was carried out following the standard protocol [9]. Each sample was dissolved in methanol at 10 mg/mL and standards i.e. quercetin, myricetin, catechin, gallic acid, apigenin, kaempferol, caffeic acid, and rutin were prepared as 50 μg/mL in methanol. All the solutions were filtered through 0.2 μm sartolon polyamide membrane filter. HPLC system was equipped with C8 analytical column and coupled with diode array DAD detector. For the analysis of poly phenols, the mobile phases were comprised as mobile phase A; acetonitrile-methanol-water-acetic acid (5:10:85:1) and mobile phase B; acetonitrile-methanol-acetic acid (40:60:1). The flow rate was maintained at 1 mL/min. Sample solution (20 μL) was injected into the column (Zorbax RX-C8 4.6 x 250 mm, 5 μm) followed by a column reconditioning for 10 min before next analysis. The gradient volume of B was 0–50% in 0–20 min, 50–100% in 20–25 min and then 100% from 25 to 30 min. The absorption of samples was recorded at 257 nm (rutin and gallic acid), 279 nm (catechin), 325 nm (caffeic acid) and 368 nm (myricetin, querecetin, kaempferol and Apeginin).

### Biological evaluation

#### Determination of total antioxidant capacity (TAC)

Phosphomolybdenum based colorimetric assay was employed to determine total antioxidant capacity and is expressed as μg equivalent to ascorbic acid per mg plant dry weight (μg AAE/mg extract) [7] with slight modification. An aliquot of 0.1 mL of each extract (4 mg/mL in DMSO) was mixed with 0.9 mL of reagent (0.6 M sulphuric acid, 28 mM sodium phosphate and 4 mM ammonium molybdate solution in H_2_O). Blank contained 0.9 mL of reagent solution and 0.1 mL of DMSO without extract; while 4 mg/mL ascorbic acid served as positive control. All tubes were kept in water bath for 90 min at 95 °C and then cooled to room temperature. A volume of 200 μL was transferred to 96 well plate and the absorbance was measured at 630 nm using microplate reader (Biotech USA, microplate reader Elx 800). A calibration curve (y = 0.0211x + 0.0920, R^2^ = 0.9911) of ascorbic acid was prepared at final concentrations of 100, 50, 25, 12.5, 6.25, 3.12 μg/mL.

### Reducing power assay

Standard potassium ferricyanide colorimetric assay was performed to estimate the reducing power potential of extracts [9]. An aliquot of 200 μL of extracts (4 mg/mL DMSO) was mixed with 400 μL of phosphate buffer (0.2 mol/L, pH 6.6) and potassium ferricyanide (1% w/v in H_2_O). The mixture was incubated for 20 min at 50 °C followed by addition of 400 μL of trichloroacetic acid (10% w/v in H_2_O) and centrifuged at 3000 rpm at room temperature for 10 min. The upper layer of solution (500 μL) was mixed with distilled water (500 μL) and 100 μL of FeCl_3_ (0.1% w/v in H_2_O). From this mixture, 200 μL was transferred to 96 well plate and absorbance of the reaction mixture was measured at 630 nm. Blank was prepared by adding 200 μL DMSO to the aforesaid reaction mixture instead of extract. A calibration curve (y = 0.037x + 0.7482, R^2^ = 0.9961) of ascorbic acid was obtained at final concentrations of 100, 50, 25, 12.5, 6.25, 3.12 μg/mL and the resultant reducing power of each sample is expressed as μg AAE/mg extract.

### DPPH free radical scavenging assay

The DPPH free radical scavenging activity of extracts was evaluated by monitoring their capability to quench the stable 2, 2-diphenyl-1-picrylhydrazyl (DPPH) free radical [7, 10, 11]. Briefly, 20 μL of four different dilutions of each sample with final concentrations of 200, 66.66, 22.22 and 7.41 μg/mL, were mixed with 180 μL of DPPH solution (9.2 mg/100 mL in methanol). After incubating the plate for 30 min at 37 °C, absorbance was recorded at 515 nm. Percent free radical scavenging activity (%FRSA) was calculated by using the formula:

$$ \%\mathrm{FRSA} = \left(1\hbox{-} {\mathrm{Ab}}_{\mathrm{s}}/{\mathrm{Ab}}_{\mathrm{c}}\right) \times 100 $$

Where Ab_s_ is the absorbance of test sample, whereas Ab_c_ is the absorbance of negative control containing the DMSO instead of sample. Ascorbic acid was used as positive control. Afterwards IC_50_ of samples with significant radical scavenging efficiency (>50%) was also calculated.

### Brine shrimp lethality assay

Lethality test was performed in a 96 well plate using brine shrimp (*Artemiasalina*) larvae as previously described (Bibi et al., 2011) with slight modification. *A. salina* eggs (Ocean star, USA) were incubated for 24-48 h under light at 30-32 °C in simulated sea water (38 g/L supplemented with 6 mg/L dried yeast) in a specially designed two-compartment plastic tray. Ten mature phototropic nauplii were harvested with the help of Pasteur pipette and transferred to each well of plate. Corresponding volume of each extract containing ≤ 1% DMSO in sea water at final concentrations of 200, 100, 50 and 25 μg/mL was transferred to the wells containing sea water and shrimp larvae. The final volume in each well was kept 300 μL. Positive and negative control wells included serial concentrations of doxorubicin and 1% DMSO, respectively. After 24 h incubation, live shrimps were counted and percentage of deaths was determined. Median lethal concentration (LC_50_) was calculated using table curve 2D v5.01 software.

### Protein kinase inhibition assay

The assay was accomplished by *Streptomyces* 85E strain according to previously documented procedure [12] with slight modification. *Streptomyces* was refreshed in sterile trypton soy broth (Merck, Germany) for 48 h and the revived culture was inoculated on petri plates containing ISP4 mineral medium. Each test sample (5 μL of 20 mg/mL as 100 μg/disc) was impregnated on 6 mm Whatman filter paper sterile disc and placed on seeded plates. The plates were incubated at 28 °C for 72 h to allow the development of hyphae. The bald zone of hyphae formation inhibition was measured with the help of vernier calliper. Extracts producing an inhibition zone ≥ 10 mm in diameter were screened to determine minimum inhibitory concentrations (MICs) at lower concentrations ranging from 50 to 3.12 μg/disc. Surfactin (5 μL of 4 mg/mL in DMSO) and DMSO impregnated discs served as positive and negative controls, respectively.

### Cytotoxicity against THP-1 leukemia cell line

The in vitro cytotoxicity evaluation of extracts against humanleukemia (THP-1) cell line (ATCC # TIB-202) was performed using standard protocol [13] with slight modifications. Briefly, leukemia cells were grown in growth medium [RPMI-1640 buffered with 2.2 g/LNaHCO_3_ and supplemented with 10% v/v heat inactivated fetal bovine serum (HIFBS); pH 7.4] in a humidified carbon dioxide incubator (37 °C, 5% CO_2_). An aliquot of 190 μL of THP-1 cells (seeding density of 1 × 10^4^ cells per mL) was transferred to each well of 96 well plate having 10 μL of sample containing 1% DMSO in PBS corresponding to final concentration of 20 μg/mL. The culture was incubated at 37 °C for 72 h in humidified CO_2_ (5%) incubator. Serial concentrations of fluorouracil and vincristine were employed as positive controls whereas 1% DMSO in PBS served as negative control. Afterwards, 20 μL of pre-filter sterilized MTT solution (4 mg/mL in distilled H2O) was added and plates were again incubated at 37 °C for 4 h in humidified CO_2_ (5%) incubator. After incubation 170 μL supernatant was removed carefully by using micropipette without disturbing colored formazan sediments. To dissolve the formazan sediments 100 μL of DMSO was added in each well, the plate was kept aside for one h to ensure full dissolution and the absorbance was measured at 540 nm using microplate reader. Samples showing more than 50% cell mortality at 20 μg/mL were further analyzed at lower concentrations i.e. 10, 5, 2.5 and 1.25 μg/mL. LC_50_ was calculated using table curve 2D v5.01 software.

### Antibacterial activity

#### Disc diffusion method

Susceptibility of extracts against bacterial species was tested according to formerly described procedure [14]. Two Gram positive (*Micrococcus luteus* ATCC # 10240 and *Staphylococcus aureus* ATCC # 6538) and three Gram negative strains (*Bordetella bronchiseptica* ATCC # 4617, *Salmonella typhimurium* ATCC # 14028 and *Enterobacter aerogenes* ATCC # 13048) were tested. The strains were cultured in nutrient broth media and incubated for 24 h at 37 °C. Sterilized deionized water was used to adjust the turbidity to 10^4^ CFU/mL by comparing with McFarland 0.5 BaSO_4_ turbidity standards. The refreshed inoculum (100 μL) was then swabbed onto Petri plates containing 20 mL nutrient agar. Test samples (5 μL of 20 mg/mL DMSO; 100 μg/disc) were infused on sterile filter paper discs (6 mm) and placed on seeded nutrient agar plate. Roxithromycin and Cefixime-USP at a concentration of 20 μg/disc and DMSO impregnated discs were included as positive and negative controls, respectively. After 24 h incubation, clear zones of growth inhibition were measured.

#### Broth micro-dilution method

The samples having ≥ 10 mm zone of inhibition (ZOI) were tested at lower concentrations using standard three-fold micro broth dilution method [15]. Test 50 μL of each bacterial suspension in suitable growth medium was added to the wells of a sterile 96-well microtitre plate already containing plant extract was serially diluted with nutrient broth to obtain a concentration ranging from 100 μg/mL to 1.11 μg/mL. The final volume in each well was 100 μL. Control wells were prepared with culture medium bacterial suspension only. The contents of each well were mixed on a microplate shaker (Hamburg, Germany) at 900 rpm for 1 min prior to incubation for 24 h. The plates were incubated for 24 h in dark at 37 °C. After incubation the plates were analyzed visually to determine the growth of bacteria in the respective well. The minimum concentration of sample showing no growth was taken as MIC (minimum inhibitory concentration).

### Antifungal activity

Antifungal activity of extracts was evaluated following previously described standard protocol [14]. The fungal strains (*Aspergillus fumigates* FCBP # 66, *Fusarium solani* FCBP # 0291, *Mucor* specie FCBP # 0300, *Aspergillus flavus* FCBP # 0064 and *Aspergillus niger* FCBP # 0198; purchased from fungal culture bank of Pakistan) were cultured on SDA. Prior to the sensitivity determination, the spores were harvested in 0.02% Tween 20 solution and turbidity was adjusted according to McFarland 0.5 turbidity standard. The 100 μL of respective harvested spores was swabbed on plates containing 25 mL sterilized SDA. Filter paper discs loaded with 5 μL of test sample (20 mg/mL DMSO; 100 μg/disc) as well as standard antifungal terbinafine (50 μg/disc) and DMSO were placed on seeded SDA plates. The plates were incubated at 28 °C for 24–48 h. Thereafter, clear zones of inhibition around discs were measured using vernier caliper.

### Antileishmanial activity

The in vitro antileishmanial evaluation of test extracts was carried out by employing MTT colorimetric assay [16]. A 6–7 days incubated culture of *Leishmania tropica* kwh 23 promastigotes was used. Concisely, parasites were grown in Medium 199 supplemented with 10% foetal bovine serum (FBS), 100 μg/mL streptomycin sulphate and 100 IU/mL penicillin G at 24 °C. An aliquot of 180 μL of promastigote culture at a pre-adjusted seeding density of 1 × 10^6^ promastigotes/mL was transferred to each well of 96 well plate having 20 μL of test samples (containing ≤ 1% DMSO in PBS). Amphotericin B (0.33–0.004 μg/mL) and 1% DMSO in PBS instead of test sample were employed as positive and negative controls, respectively. The culture plate was incubated at 24 °C for 72 h after that 20 μL of pre-filter sterilized MTT solution (4 mg/mL in distilled H_2_O) was added and plates were again incubated at 24 °C for 4 h. After incubation supernatant was removed carefully without disturbing colored formazan sediments. To dissolve the formazan sediments 100 μL of DMSO was added in each well, the plate was kept aside for 1 h to ensure full dissolution and the absorbance was measured at 540 nm using microplate reader. Samples showing more than 50% cell mortality at 100 μg/mL were further analyzed at lower concentrations i.e. 33.3, 11.1, 3.7 and 1.23 μg/mL.LC_50_ was calculated by using table curve 2D v5.01 software.

### Statistical analysis

All the experiments were carried out in triplicate. The data were presented as mean ± standard deviation (SD). SPSS Ver. 21 software was used for Post Hoc Multiple Comparison test in One Way ANOVA and IC_50_ was determined by using table curve software 2D Ver. 4.

## Results and discussion

*R. punjabensis* stewart leaves and stem extracts were prepared in varied polarities solvents and maximum extract yield was obtained by EtOH + EtOAc (6.11%) and CHCl_3_ (5.49%) from leaf and stem parts, respectively (Table 1). n-Hex extract exhibited the minimum yield (1.25 and 0.56% from leaf and stem parts, respectively). The difference in extraction yield from different plant parts in similar solvents depicts organ specific accumulation of secondary metabolites in *R. punjabensis* stewart. The extraction is also based on solubility of chemical constituents on polarity basis, it essential to study about the relationship between the extraction methodapplied and their physiochemical properties to substances to be extracted (Medziga et al., 2010).

**Table 1:** Percent extract recoveries of leaf and stem of *R. punjabensis* using different solvents

Samples	%Extract Recovery	
	Leaf	Stem
n-hex	1.25 ± 0.82	0.56 ± 0.02
CHCl3	2.44 ± 0.15*	5.49 ± 0.22***
Acet	4.69 ± 1.04	2.00 ± 0.43
Acet + EtOAc	5.83 ± 1.21***	2.05 ± 0.08*
EtOAc	3.71 ± 0.09	2.08 ± 0.87
EtOH	3.89 ± 1.08	2.25 ± 0.07
MeOH	4.90 ± 1.03**	3.34 ± 0.23**
MeOH+ CHCl3	5.29 ± 1.54***	2.44 ± 0.04
EtOH+ CHCl3	3.53 ± 1.21	2.07 ± 0.14*
EtOH + EtOAc	6.11 ± 1.09***	2.48 ± 0.07
MeOH + EtOAc	5.20 ± 1.23	2.83 ± 0.98*

Values (mean ± SD) are average of three samples of each plant part, analyzed individually in triplicate (*n* = 1x3 x 3). Data is presented as highly significant***, slightly significant**, significant* at *p* < 0.05. *n*-hexane: *n*-hex, chloroform: CHCl_3_, acetone: Acet, ethyl acetate: EtOAc, ethanol: EtOH, methanol: MeOH, ethanol + chloroform: EtOH + CHCl_3_, methanol + chloroform: MeOH + CHCl_3_, acetone + ethyl acetate: Acet + EtOAc, ethanol + ethyl acetate: EtOH + EtOAc, methanol + ethyl acetate: MeOH + EtOAc

**Fig. 1:**
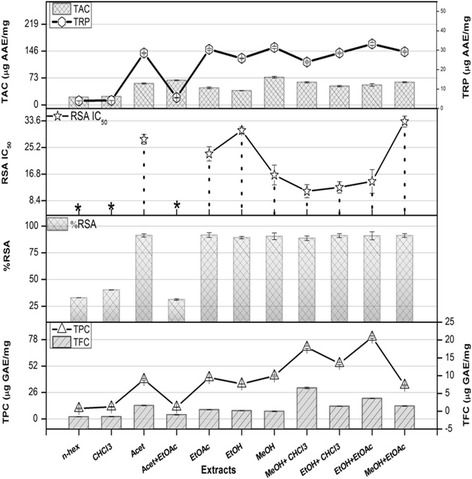
TPC (Total phenolic content μg GAE/mg extract), TFC (Total flavonoid content μg QE/mg extract), %RSA (radical scavenging activity), TAC (Total antioxidant capacity μg AAE/mg extract) and TRP (Total reducing power μg AAE/mg extract) potential of *R. punjabensis* Leaf.*IC50> 200 μg/ml Values are presented as mean ± Standard error from triplicate investigation

**Fig. 2:**
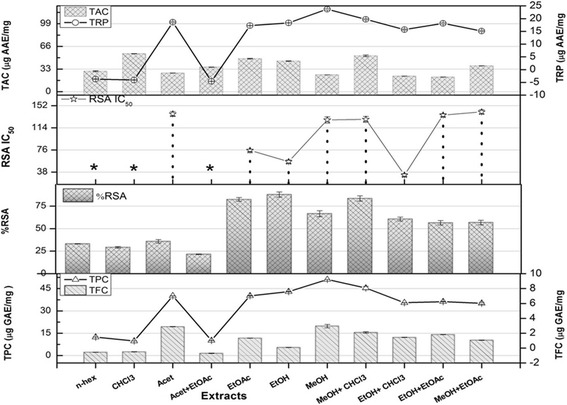
TPC (Total phenolic content μg GAE/mg extract), TFC (Total flavonoid content μg QE/mg extract), %RSA (radical scavenging activity), TAC (Total antioxidant capacity μg AAE/mg extract) and TRP (Total reducing power μg AAE/mg extract) potential of *R. punjabensis* Stem.*IC50> 200 μg/ml Values are presented as mean ± Standard error from triplicate investigation

Isolation of natural compounds, in particular, plant-derived antioxidants has gained interest of scientists to treat diseases by reducing oxidative stress [17]. The Highest TPC (80.5 ± 2.18 μg GAE/mg extract) was quantified in MeOH+ CHCl_3_ extract of leaves, followed by the EtOH+ EtOAc(75.5 ± 1.75 μg GAE/mg extract) (Fig. 1). In case of stem, significant quantity was noticed in MeOH extract (51.1 ± 1.14 μg GAE/mg extract) followed by MeOH+ CHCl_3_ (45.4 ± 1.11 μg GAE/mg extract) (Fig. 2). The phenolic contents in leaf were found higher in moderately polar solvents. This is in agreement with previous studies,where polar solvent are reported efficient for the extraction of phenolics in other *Rhus* species [18, 19]. Significant TFC (30.50 ± 1.11 μg QE/mg extract) was found in MeOH+ CHCl_3_ leaf extract, followed by EtOH+ EtOAc extract (20.1 ± 0.75 μg QE/mg extracts); while in case of stem highest TFC was found in MeOH (19.5 ± 0.75 μg QE/mg extract). Antioxidant capability of phenols attribute to the presence of methoxy, hydroxyl, double bond conjugation or ketonic group in a phenolic molecule [4]. In a previous study, flavonols likequercetin, myricetin, and kaempferol have been identified in EtOAc and MeOH extracts of *Rhus coriaria L* leaves along with gallic acid, methyl gallate, *m*-digallic acid, and ellagic acid as a part of tannins [20]. Flavonoids produce antioxidative effects and also are potent neuro protective and anti-inflammatory effects as depicted by *R. verniciflua L* bark extract [21]. It can be depicted that as *R. punjabensis* has significantly high amount of flavonoids in its extracts, it may have the above mentioned flavonoids in it For the purpose, quantification of some flavonoids and phenols in extract was carried out by reverse phase HPLC. Significant amount of flavonoid glycosides, flavone aglycones and gallic acid were quantified in some extracts. Among the leaf extracts, substantial amount of rutin was detected in the EtOH extract (0.01 μg/mg extract) and MeOH extract (0.3 μg/mg extract). Maximum content of gallic acid (0.6 μg/mg extract) and catechin (0.8 μg/mg extract) were quantified in EtOH + CHCl_3_ leaf extract. Rutin (0.4 μg/mg extract) and apigenin (0.6 μg/mg extract) were also significantly present in MeOH+ CHCl_3_stem extract (Table 2). Chromatographic fingerprinting by HPLC is considered a simple, reproducible, sensitive and reliable method for chemical profiling of analytes [9]. The flavonoids under investigation possess significant biological activities. For example rutin contributes to antibacterial and antioxidant properties, while gallic acid and apigenin induce autophagy in leukemia cells, which proves their chemo preventive and anticancer role [22]. Previously existence of rutin and other flavonoids establish significant correlation with momentous antioxidant and antibacterial potential of *R. verniciflualaquer*. Flavonoids such as myricetin, quercetin, kaempferoletc has been reported in *R. coriaria. L* [23]. and bear significant antioxidant and cytotoxic activities [24, 25]. The previous reports on other *Rhus spp.* are in accordance with the current investigation.

**Table 2:** Chemical profiling of stem and leaf extracts of *R. punjabensis* using HPLC-DAD

Samples	Polyphenols (μg/mg DW)				
	Gallic acid	Rutin	Caffeic acid	Catechin	Apigenin
Leaf					
n-hex	__	__	__	__	__
CHCl3	__	__	__	__	__
Acet	__	__	__	__	__
Acet + EtOAc	__	__	__	__	__
EtOAc	__	__	__	__	__
EtOH	__	0.1^d^	__	__	__
MeOH	0.5^c^	0.3^ab^	__	__	__
MeOH+ CHCl3	0.6^b^	__	__	0.3^ab^	__
EtOH + CHCl3	__	__	__	__	__
EtOH + EtOAc	__	__	__	__	__
MeOH + EtOAc	__	__	__	__	__
Stem					
n-hex	__	__	__	__	__
CHCl3	__	__	__	__	__
Acet	__	__	__	__	__
Acet + EtOAc	__	__	__	__	__
EtOAc	0.1	__	__	__	__
EtOH	__	__	__	__	__
MeOH	0.8^a^	__	__	__	__
MeOH+ CHCl3	0.4^c^	0.4^c^	__	__	0.6^b^
EtOH+ CHCl3	0.2^e^	__	__	__	0.3^ab^
EtOH + EtOAc	__	__	__	__	__
MeOH + EtOAc	__	__	__	__	__

__ = not detected. Myricetin, Kaempferol,Quecetin were not detected in all extracts. LSD and HSD applied all the data. The small alphabates marked on values show significantly difference within mean values within cloumn using LSD test at *p* <0.05

Significant antioxidant capacity (74.5 ± 2.25 μg AAE/mg extract) was observed in MeOH extract of leaf followed by the Acet + EtOAc extract (66.9 ± 1.11 μg AAE/mg extracts) (Fig. 1). Among stem extracts (Fig. 2), the highest TAC (54.4 ± 1.12 μg AAE/mg extract) was found in CHCl_3_ extract followed by MeOH + CHCl_3_ extract (52.3 ± 1.16 μg AAE/mg extract). The plants have the major potential for the natural antioxidants, phytochemicals and secondary metabolites [26]. Maximum activities shown by EtOH and EtOH+ CHCl_3_ can be accredited to the presence of gallic acid, rutin and catechin as indicated by HPLC in this report. Phenolic compounds disrupt chain oxidation reactions by donation of a hydrogen atom or chelating metals. The high amounts of phenolic compounds indicate high antioxidant and reducing power activities [27]. Positive correlation has been found among reducing power, antioxidant, total phenolic, and total flavonoids contents specifically in moderately polar solvents, that is in good agreement with previously documented reports [28].

In TRP assay, yellow color of test solution changes to green or blue depending on the reducing power of samples. The Highest reducing power potential (165.4 ± 2.29 μg AAE/mg extract) was observed by EtOH + EtOAc leaf extract followed by MeOH extract (68.94 ± 3.76 μg AAE/mg extract) (Fig. 1). In case of stem, the highest TRP was quantified in MeOH extract (120.2 ± 1.12 μg AAE/mg extracts) followed by MeOH + CHCl_3_ extract (Fig. 2). Previous studies depict that comparatively polar extracts show high reducing power [29]. The presence of rutin in MeOH + CHCl_3_ stem extract can be the possible cause of its maximum reducing power as interaction of rutin with superoxide ion and ferrous ions can cause effective inhibition of iron ion-dependent lipid peroxidation systems [30]. However, absence of quantified flavonoids in MeOH + CHCl_3_ leaf extract by HPLC-DAD analysis suggests the probable role of additional flavonoids; require further exploration.

DPPH free radical scavenging potential is based on the ability of antioxidants to decolorize 2, 2-diphenyl-2-picryl-hydrazyl. Among all the extracts analyzed, the IC_50_ ranged from 11.4 to 33.4 μg/mL (Fig. 1). MeOH + EtoAc leaf extract showed highest DPPH scavenging activity (IC_50_) inhibitory concentration (IC_50_ 11.4 μg/mL). In case of stem extract, considerable DPPH scavenging activity was observed by EtOH + CHCl_3_ extract (IC_50_ 33.8 μg/mL). Studies have demonstrated that free radical scavenging activities might be attributed to the presence of phenolics as a good correlation has been found between the total phenolic content and free radical scavenging activity (R^2^ = 0.878).*R. vericiflua stroke* has been reported that exhibit marked antioxidant and antitumor properties, and also antioxidant activity on the Fe^+2^ induced linoleic acid peroxidation [24]. In traditionally the Most of the research performed on *Rhus* extracts has examined antioxidant activity. The work to date has been focused on various number of species (*R. verniciflua* and *R. succedanea* in Asia, *R. hirta* in northeastern North America) and *R. coriaria* in the Mediterranean/Middle East. More breadth in worldwide species is required to better understand the potential of *Rhus* as a commercial source of natural antioxidants [31].

Brine shrimp lethality assay is a prescreening technique for determination of cytotoxic potential of natural products [32]. Stem and leaves extracts of *R. punjabensis* prepared in different solvent systems showed noteworthy activities (Table 3) with LC_50_(50% lethal concentration) ranging from 36.7 to 93.3 μg/mL. n-hex leaf extract showed highest cytotoxic effect against brine shrimps (LC_50_ 36.7 ± 0.56 μg/mL) followed by CHCl3 extract (LC_50_38.5 ± 0.65 μg/mL). The degree of lethality is normally presumed directly proportional to the potency of extract. The results suggest that use of moderately polar solvent for extraction is a better option for isolation of compounds with cytotoxic capability, rather than a highly polar or non-polar solvent. The results of cytotoxic assay in current study showed lower values of LC_50_ than previously reported in *R. lancea L* leaves (600 μg/mL). Brine shrimp cytotoxicity assay is widely used as initial procedure for exploration of antimicrobial, antitumor, antifungal, antimalarial, molluscicidal, larvicidal and insecticidal activities [33]. In recent years, the Chinese herbal medicine has been used the allied species of *Rhus* discussed widely as a new alternative for carious diseases. The results of this assay depict that *R. punjabensis* may have more hidden potential for the discovery of bioactive compounds in addition to the present investigation.

**Table 3:** Cytotoxic, antileishmanial and protein kinase inhibitory activities of *R. punjabensis* leaf and stem extracts

Samples	Brine shrimp cytotoxicity (μg/ml)		Cytotoxicity against leishmanial promastigotes (μg/ml)		THP-1 cytotoxicity (μg/ml)		Protein kinase inhibition (μg/disc)	
	% Mortality	LC_50_	% Mortality	IC_50_ (μg/mL)	% Mortality	IC_50_	*Diameter (mm) at 100 μg/disc	
	200		100		10		Clear zone	Bald zone
Leaf								
n-hex	100 ± 4.10	36.7 ± 0.56	98.2 ± 3.12	15.78 ± 0.77	--	--	7 ± 0.51	--
CHCl3	98 ± 3.11	38.5 ± 0.65	90.7 ± 3.05	18 ± 0.18	64.2 ± 0.71	7.8*** ± 0.61	6 ± 0.62	--
Acet	96 ± 3.00	52.2 ± 0.66	95.3 ± 3.18	21.60 ± 0.25***	26.1 ± 0.10	>10	__	9 ± 0.7
Acet + EtOAc	92 ± 2.75	58.7 ± 0.71***	75 ± 1.19	35.43 ± 0.41	61.9 ± 0.71	8.1** ± 0.71	8 ± 0.73	--
EtOAc	86 ± 1.31	64.5 ± 0.78	72 ± 1.22	64.50 ± 0.62**	--	--	--	6 ± 0.56
EtOH	82 ± 1.16	62.7 ± 0.81**	66.4 ± 0.75	75.40 ± 1.17	--	--	--	12.5 ± 1.71***
MeOH	65 ± 0.75	86.2 ± 1.18e	63.6 ± 0.66	81.60 ± 1.12	40.4 ± 0.61	>10	--	13.5 ± 1.11***
MeOH+ CHCl3	67 ± 0.71	88.1 ± 1.51	69.3 ± 0.80	77.75 ± 1.23*	35.7 ± 0.21	>10	--	11 ± 0.87**
EtOH+ CHCl3	62 ± 0.72	87.9 ± 1.30	54.6 ± 0.57	97.8 ± 3.11*	**--**	--	--	11 ± 0.88**
EtOH + EtOAc	68 ± 0.81	82.3 ± 1.11*	68.2 ± 0.77	57.41 ± 0.75	--	--	6.5 ± 0.11	--
MeOH + EtOAcet	57 ± 0.55	93.2 ± 1.75	72.7 ± 1.01	54.55 ± 0.71	--	--	6.6 ± 0.12	--
Stem								
n-hex	100 ± 4.13	39.6 ± 0.65	62.6 ± 0.80	75.74 ± 1.10**	--	--	8 ± 0.75	--
CHCl3	98 ± 2.16	43.9 ± 0.56b	52.8 ± 0.56	78.72 ± 1.19	54.7 ± 0.71	8.4** ± 0.72	9.5 ± 0.98	--
Acet	87 ± 1.33	55.1 ± 0.60*	48.1 ± 0.46	>100	52.3 ± 0.70	8.9* ± 0.88	6.5 ± 0.65	--
Acet + EtOAc	76 ± 1.18	62.7 ± 0.77***	46.6 ± 0.41	>100	--	--	6.5 ± 0.66	--
EtOAc	78 ± 1.19	59.5 ± 0.56**	49.2 ± 0.51	>100	--	--	8 ± 0.85	--
EtOH	72 ± 1.15	64.4 ± 0.68	56.7 ± 0.80	97.73 ± 3.15***	--	--	7.5 ± 0.62	--
MeOH	75 ± 1.17	68.7 ± 0.71**	52.2 ± 0.90	95.58 ± 1.10	--	--	--	12.5 ± 1.12***
MeOH+ CHCl3	62 ± 0.72	68.3 ± 0.72	28.4 ± 0.07	>100	16.6 ± 0.11	>10	--	8 ± 0.66
EtOH+ CHCl3	45 ± 0.09	>200	26.7 ± 0.05	>100	--	--	--	10 ± 0.75**
EtOH + EtOAc	42 ± 0.07	>200	18.7 ± 0.08	>100	--	--	--	9 ± 0.96
MeOH + EtOAc	48 ± 0.07	>200	14.4 ± 0.07	>100	--	--	7 ± 0.70	--
Doxorubicin	100 ± 4.10	5.75 ± 0.07						
Flourouracil					99.6 ± 4.19	5.1 ± 0.11		
Vincristine					98.1 ± 3.17	8.1 ± 0.92		
Surfactin								17 ± 1.02
Amphotercin-B			100 ± 4.01	1.36 ± 0.11		--	--	--
1% DMSO in PBS/sea water	--		--		--			
DMSO								--

*Zone of inhibition including the diameter of disc (5 mm). Data was analyzed individually in triplicate (*n* = 1x3). Data is presented as highly significant***, slightly significant**, significant* at *p* <0.05. --: no activity. LSD and HSD applied in all the data

Significant protein kinase inhibition was observed by MeOH extract of leaves (50 μg/disc). While in case of stem, the highest activity was found by MeOH extract (Table 3). Overall maximum protein kinase inhibitory potential was found the polar organic solvents. Researchers around the world have shown interest in the identification of kinase inhibitors which can lead to the development of new drugs for chemopreventive measures [34]. Deregulation of protein kinases is a key factor that play vital role in the pathogenesis of disease [35]. Point mutations in tyrosine kinases and phosphatidylinositol 3-kinase catalytic (PI3KCA) subunit in colorectal and gastric cancer [36] and epidermal growth factor receptor activating kinase mutation in glioblastoma [37] are few examples of such deregulations. Protein kinase inhibitors block the aerial hyphae formation of *Streptomyces* sp., thus may be hypothesized to inhibit the cancer cell proliferation [12].

The in vitro cytotoxic values of extracts against THP1human leukemic cell line are presented in Table 3. CHCl_3_ extract of leaves and stem showed good anticancerous effect (LC_50_7.8 ± 0.61 μg/mL and 8.4 ± 0.72 μg/mL, respectively), which is comparable to standard drugs 5-florouracil and vincristine (LC_50_ 5.1 μg/mL and 8.1 μg/mL, respectively). The cytotoxic activity against cell lines might be attributed to the presence of molecules which have the ability to modify the signal transduction pathways; flavonoids responsible for inhibitory effects on protein kinases and some transcriptional factors [38], protein tyrosine kinase inhibitor, molecules that cause cell cycle arrest, and apoptosis by a p53-dependent mechanism [39]. The compounds isolated from *R. succedanea* L have shown significant cytotoxic activity against five cancer cell lines including cervix epithelioid carcinoma (HeLa), hepatoma cell line (Huh7), colorectal cancer cell line (HCT116), colon adenocarcinoma (LoVo), and rat C6 glioma cells [40]. The brine shrimp lethality assay also showed good relationship with cytotoxic activities against THP1 cell line in the current exploration. The results are in consistent with the literature where the significant cytotoxic, anticancer activity against carcinogenic Cdc25 phosphatases was unveiled by *R. chinensis Mill* [41] and *R. verniciflua stokes* against mouse embryonic primary hepatic cells (MPHC), embryonic normal hepatic cell line (BNL CL.2), and SV40-mediated transformed cell line (BNL SV A.8) [42, 43]. The similarity in the results of some assays suggest that *R. punjabesis* may also have the similar potential for drug discovery as the other allied species of this genus.

The antibacterial activity of extracts from leaves and stem parts of *R. Punjabensis*are shown in Table 4. The extracts producing a growth inhibitory zone of ≥10 mm in agar disc diffusion assay are considered active and further evaluated for MIC determination through broth micro dilution method. All the extracts were significantly effective against tested bacterial strains. Among all, *M. Luteus* and *S. Typhimurium* strains were found most susceptible by the Acet and EtOAc leaf extract, producing zones of inhibition of 18 ± 1.5 and 22 ± 1.5 mm with MIC of 11.11 and 1.11 μg/mL, respectively. MeOH + CHCl_3_ stem extract depicted maximum growth inhibition zone (16 ± 1.7 mm) with MIC 11.1 and 33.33 μg/mL against *M. luteus*and *P. aeruginosa,* respectively*.* The antimicrobial properties of herbal plants largely depends upon solvent used, organism tested and plant part used. Various species of *Rhus* genus have been documented to possess the antibacterial activities [4]. The allied species of *Rhus;* R. glabra is traditionally used by native peoples of North America in the treatment of bacterial diseases such as syphilis, gonorrhea, dysentery, and gangrene. R. coriaria which grows wild in the region from the Canary Islands through the Mediterranean region to Iran and Afghanistan [44]. Now a day, world is facing greatest problem of resistance against the conventional antibiotics from the microbial sources. This has largely increased the surge for the discovery of new antimicrobial from the other sources. In the present study, tremendous antibacterial potential of *R. punjabensis* suggests it a good alternate to the antibiotics. *Rhus* extracts are most notable for their antimicrobial activities [3]. It also propose an in depth study of this plant with complete focus on its antibacterial potential to isolate the lead compounds.

**Table 4:** Antibacterial activities of leaf and stem extracts of *R. punjabensis*in terms of zone of inhibition (ZOI) and their MICs

Extracts	Zone of inhibition (mm) at 100 μg/disc and MIC (μg/mL)									
	*P. aeruginosa*	MIC	*S. typhimurium*	MIC	*M. luteus*	MIC	*B. bronchiseptica*	MIC	*S. aureus*	MIC
Leaf										
n-hex	--	--	--	--	--	--	13 ± 1.24***	33.3^c^	12 ± 1.17*	33.3^c^
CHCl3	--	--	--	--	--	--	08 ± 0.49	--	11 ± 1.16	100
Acet	10 ± 1.15	100	13 ± 1.29*	33.3	18 ± 1.85***	11.11^ab^	14 ± 1.23**	33.3^c^	08 ± 0.66	--
EtOAc	10 ± 1.14	100	22 ± 2.25***	1.11^a^	14 ± 1.12	11.11^ab^	11 ± 1.19	100	12 ± 1.26	100
EtOH	06 ± 0.56	--	10 ± 1.19	100	13 ± 1.56	33.3^c^	12 ± 1.11*	100	11 ± 1.18	100
MeOH	14 ± 1.66*	33.3^ab^	--	--	--		06 ± 0.25	--	18 ± 19.6	11.11^ab^
EtOH+ CHCl3	10 ± 1.11	100	10 ± 1.18	100	18 ± 1.65***	11.11^ab^	16 ± 1.59**	33.3^c^	08 ± 0.77	--
MeOH+ CHCl3	--	--	13 ± 1.55	33.3^c^	10 ± 1.13	33.3^c^	09 ± 0.98	--	09 ± 0.85	--
EtOH + EtOAc	12 ± 1.55	33.3^ab^	18 ± 2.98***	11.3^ab^	15 ± 1.44**	11.11^ab^	10 ± 1.12	100	13 ± 1.26**	33.3
MeOH + EtOAc	13 ± 1.65*	33.3^ab^	15 ± 1.12**	11.11^cb^	15 ± 1.73**	11.11^ab^	10 ± 1.12	100	08 ± 0.66	--
Acet + EtOAc	15 ± 1.84**	11.11^a^	14 ± 1.51*	11.11^cb^	20 ± 2.93***	1.11^a^	11 ± 1.11	100	13 ± 1.56	33.3^c^
Stem										
n-hex	--	--	13 ± 1.37	100	10 ± 1.17	--	12 ± 1.24	100	14 ± 1.97**	33.3^c^
CHCl3	05 ± 0.78	--	10 ± 1.14	100	--	--	11 ± 1.14	--	06 ± 0.57	--
Acet	11 ± 1.27	100	15 ± 1.58**	33.3	11 ± 1.15	100	13 ± 1.67**	33.3^c^	10 ± 1.16	--
EtOAc	11 ± 1.26	100	11 ± 1.71	100	14 ± 1.29**	33.3^c^	13 ± 1.68	--	10 ± 1.17	--
EtOH	10 ± 1.17	100	10 ± 1.17	100	10 ± 1.15	--	13 ± 1.74	--	15 ± 1.53**	33.3^c^
MeOH	--	--	07 ± 0.88	--	--	--	10 ± 1.14	--	12 ± 1.25	--
EtOH+ CHCl3	--	--	07 ± 0.79	--	12 ± 1.19	100	12 ± 1.12	--	10 ± 1.17	--
MeOH+ CHCl3	16 ± 1.97	33.3^ab^	07 ± 0.89	--	16 ± 1.67**	11.1^ab^	08 ± 0.41	--	11 ± 1.27	--
EtOH + EtOAc	13 ± 1.10**	33.3^ab^	12 ± 1.10	100	11 ± 1.27		15.5 ± 1.97**	11.11^ab^	11 ± 1.21	--
MeOH + EtOAc	08 ± 0.98		10 ± 1.55		12 ± 1.37	100	09 ± 0.77	--	08 ± 0.89	--
Acet + EtOAc	11 ± 1.81	100	12 ± 1.73	100	11 ± 1.21	--	10 ± 1.18	--	08 ± 0.88	--
Cefixime	26 ± 2.98	1.1	25 ± 2.93	1.1	24 ± 2.71	1.1	24 ± 2.92	1.1	23.5 ± 2.71	1.1
Roxithromycin	26.5 ± 2.95	0.1	25 ± 2.53	0.1	26.5 ± 2.84	0.1	26 ± 2.85	0.1	25 ± 2.32	0.1

Values (mean ± SD) are average of three samples of each plant extract, analyzed individually in triplicate (*n* = 1x3). Means difference is highly significant*** slightly significant**, and significant* at *p* < 0.05. The MIC values within the column are statistically analyzed using LSD test at *p* <0.05. The same alphabates represent non-significant difference -- = No activity in antibacterial assay or not active (zone ˂ 10 mm) for MIC determination

The highest zone of inhibition (14 mm), in case of antifungal activity was found by MeOH extract of leaf against *A. flavus* (MIC 50 μg/disc) followed by Acet extract of leaf against *A. fumigatus*, while stem extracts showed comparatively less activities against all fungal strains (Table 5). None of the stem and leaves extract was active against *F. solani* and *A. niger.* In previous studies, moderate activity of *R. glabra. L.* branches extract has been reported against some fungal strains [45]. *R. coriaria. L.* extracts did not show prominent activity against *Candida albicans* and *Candida tropicalis* [46]. Our results are in agreement with previous studies regarding the use of comparatively polar solvents for better extraction of antimicrobial compounds [47–53].

**Table 5:** Antifungal activity of leaf and stem extracts of *R. punjabensis*tested against filamentous fungi

Samples	*Diameter of growth inhibition zone at 100 μg/disc				
	*A. fumigatus*	*Mucor*species	*A. niger*	*F. solani*	*A. flavus*
Leaf					
n-hex	--	11 ± 0.8	--	--	--
CHCl3	--	--	--	--	--
Acet	13 ± 1.21***	--	--	--	--
Acet + EtOAc	--	--	--	--	11.5 ± 1.55*
EtOAc	7.5 ± 0.74	--	--	7 ± 0.77	--
EtOH	--	09 ± 0.74	--	--	09 ± 0.95
MeOH	--	--	--	--	14 ± 1.7***
MeOH+ CHCl3	12 ± 1.17**	--	--	--	--
EtOH+ CHCl3	--	--	--	--	11.2 ± 1.6*
EtOH + EtOAc	--	--	--	--	10 ± 1.3
MeOH + EtOAcet	--	--	--	--	11 ± 1.9*
Stem					
n-hex	--	--	07 ± 0.57	--	--
CHCl3	--	7.5 ± 0.74	--	--	--
Acet	--	7.5 ± 0.84	--	--	--
Acet + EtOAc	--	10 ± 1.16	--	--	09 ± 0.86
EtOAc	--	10.5 ± 1.17	--	--	11 ± 1.15*
EtOH	_	--	--	--	--
MeOH	--	7.5 ± 0.97	--	--	--
MeOH+ CHCl3	--	7.5 ± 0.84	--	--	--
EtOH+ CHCl3	--	-	--	--	9.5 ± 0.75
EtOH + EtOAc	--	7.5 ± 0.75	--	--	--
MeOH + EtOAcet	--	08 ± 0.85	--	--	--
Terbinafine	25.5 ± 2.25	24 ± 2.52	23 ± 2.81	25 ± 2.84	25 ± 2.94
DMSO	--	--	--	--	--

*Zone of inhibition including the diameter of disc (5 mm). In each disc, the sample size was 100 μg per disc (5 μl) in disc diffusion assay. --: no activity. Means difference is highly significant*** slightly significant**, and significant* at *p* < 0.05. Values are presented as mean ± standard error from triplicate investigation

n-hex leaf extract exhibited significant antileishmanial potential with LC_50_ 15.78 ± 0.77 μg/mL, followed by the CHCl_3_leaf extract (LC_50_18 ± 0.18 μg/mL) while in case of stem extract highest activity was observed by n-hex (LC_50_75.74 ± 1.10^**^μg/mL). These results are differing to findings in that polar extract (methanol) of *R. aucheri* Boiss and *R. retinorrhaeasteud. ex. A* were found more active against leishmanial promastigotes [54, 55]. The development of resistance to the first line drugs and worldwide scattering of leishmaniasis has led to the increased demand of new therapies for the abolition of this disease necessitating further research in this aspect [56].

The research efforts on *Rhus* extracts indicate a promising potential for the plant family to provide renewable bioproducts with the following desirable bioactivities; antifungal, antiinflammatory, antimicrobial, antimalarial, antioxidant, cytotoxic, hypoglycaemic, and anticancer. As well, the bioactive components can be extracted from the plant material using environmentally benign solvents [3].

## Conclusions

The phytochemical and *invitro*biological potential of different solvent extracts of *R. punjabensis* leaves and stem is investigated. The findings of current study support the conception of utilization of multiple solvent systems for complete phytochemical and biological profiling of plants. The results indicate that different organic solvent extracts possesses diverse range of biological activities. Polar solvent extracts may be the potential source of phytochemicals provoking highly significant antioxidant capability as well as protein kinases inhibitor. Similarly moderately polar extract of this plant is remarkably effective against leishmania, brine shrimps and THP1 human leukemia cell line which signifies its cytotoxic potential. Consequently this plant can be used as a potent source of antioxidants, anticancerous and antimicrobial agents. Moreover all the reported activities require bioactivity guided isolation of corresponding extract to isolate the active compounds responsible for the reported biological activities.

## Abbreviations

DMSO: Dimethyl sulfoxide
DPPH: 2, 2-diphenyl-1-picrylhydrazyl
FRSA: Free radical scavenging activity
IC_50_: 50% inhibitory concentration
LC_50_: Lethal concentration causing 50% mortality
MIC: Minimum inhibitory concentration
TAC: Total antioxidant capacity
TFC: Total flavonoid contents
TPC: Total phenolic contents
TRP: Total reducing power
ZOI: Zone of inhibition
